# The functional results of tibial shaft fractures treated with intramedullary nail compressed by proximal tube

**DOI:** 10.1007/s11751-016-0242-x

**Published:** 2016-02-02

**Authors:** Ahmet Adnan Karaarslan, Nihat Acar, Hakan Aycan, Erhan Sesli

**Affiliations:** Orthopedics and Traumotology Department, Faculty of Medicine, Şifa University, Sanayi St. No: 7, 35040 Bornova, Izmir Turkey; Orthopedics and Traumotology Department, Ozel Gazikent Medical Center, Ulastirma St., 35410 Gaziemir, Izmir Turkey

**Keywords:** Tibial fractures, Fracture fixation, Compression nail, Fracture nonunion

## Abstract

Nailing of tibial shaft fractures is considered the gold standard surgical method by many surgeons. The aim of this retrospective study was to investigate and compare the clinical outcome of tibial shaft fractures treated with intramedullary nails compressed by proximal tube and conventional intramedullary interlocking nails. Fifty-seven patients with tibial shaft fractures, treated with intramedullary nails compressed by proximal tube (*n* = 32) and the conventional interlocking nails (*n* = 25), were reviewed. All fractures except for one were united without any additional surgical intervention in the proximal compression tube nail group, whereas in the conventional interlocking nail group, six patients needed dynamization surgery (*p* = 0.005) and three cases of nonunion were recorded. In the proximal compression tube nail group, faster union occurred in 20 ± 2 (16–24) weeks (mean ± SD; range) without failure of locking screws and proximal nail migration, whereas in the conventional interlocking nail group, union occurred in 22 ± 2.5 (17–27) weeks (*p* = 0.001) with two failures of locking screws and two proximal nail migration. The proximal compression tube nail system is safer than the conventional nailing methods for the treatment for transverse and oblique tibial shaft fractures with a less rate of nonunion, proximal locking screw failure and proximal nail migration.

## Introduction

Intramedullary interlocking nails are very commonly used in trauma practice for tibial fractures; however, conditions such as nonunion, reoperations to dynamize the nail, interlocking screw failure, and proximal nail migration are still frequently encountered problems [1, 2].

**Fig. 1:**
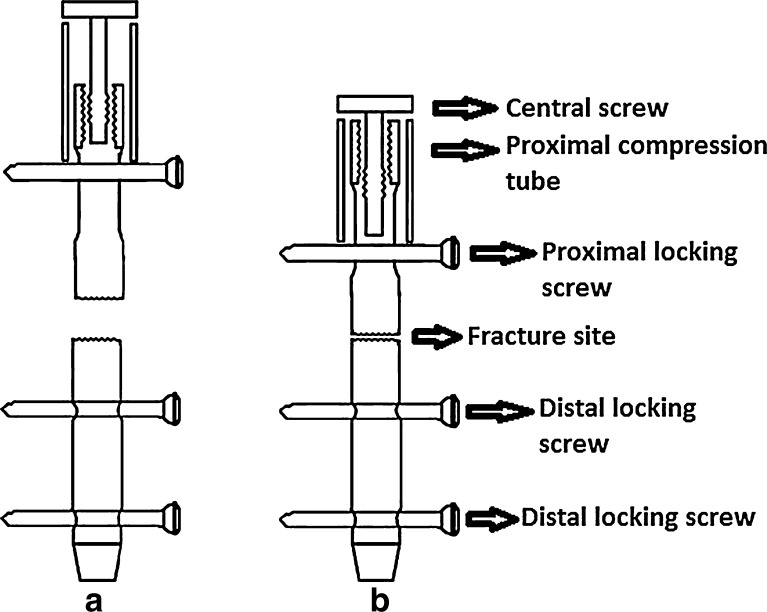
**a** Nail compressed by proximal tube before compression. **b** After tube compression

In surgical treatment of tibial shaft fractures, compression of interlocking nails provides more stability [3–7]. However, screw deformation and subsequent early locking screw failure may occur as a result of the excessive compression effect [3, 8–10]. Inter-fragmentary compression, shifting of oblique fractures, and inter-fragmentary bone resorption had been reported by some studies to cause anterior knee pain [11, 12]. We have introduced a proximal compression tube nail exerting its compression effect through a proximal tube which in turn creates a less deforming stress on the proximal locking screw and potentially minimizes proximal nail migration (Fig. 1). When compression is initiated by the proximal tube system which exerts its compression effect through a two separate points of a 13 mm in width, the bending stress will be disseminated through the screw body which in turn results in a less proximal locking screw bending failure. When compression of the fracture site is exerted by the proximal tube system, the total length of the nail will decrease. Reduction in the nail length reduces the risk of the proximal nail migration effect which frequently occurs in conventional nailing systems.

The aim of this retrospective study was to assess and compare the functional results of tibial shaft fractures operated by two methods, intramedullary nail compressed by proximal tube system and the conventional interlocking nails.

## Patients and methods

In the period from February 1997 to April 2002, 82 patients with tibial shaft fractures had been surgically treated with either conventional interlocking nails (produced by Tıpsan medical devices company, İzmir, Turkey) or intramedullary nails compressed by proximal tube system (produced by Tıpsan medical devices company, İzmir, Turkey). Patients with conditions that may delay bone healing like open tibial fractures, diabetes mellitus, and smoking were excluded from this study. Patients with postoperative complications such as neurovascular injuries, compartment syndrome, and infections were also excluded from this study. Transverse, oblique (AO/ASIF 42-A2, A3), and some spiral (AO/ASIF 42-A1, suitable for stable reduction) tibial shaft fractures were included in the study. A total of 57 patients were involved in the study and were followed up for at least 2 years (Table 1).

**Table 1:** Demographic data of patients involved in this study

	The nail compressed by tube (*n* = 32)	Conventional interlocking nail (*n* = 25)	*p* value
Gender	20 males, 12 females	17 males, 8 females	Chi-square test *p* = 0.441
Age, mean ± SD (range) Year	37.2 ± 12 (20–68)	35.1 ± 11.5 (19–69)	*t* test *p* = 0.507
Injury: falls from high	21 (66 %)	16 (64 %)	Chi-square test *p* = 0.559
Injury: motor vehicle crushes	11 (34 %)	9 (36 %)	Chi-square test *p* = 0.559
Fracture location Middle segment	20 (62 %)	13 (52 %)	Chi-square test *p* = 0.745
Distal segment	7 (22 %)	6 (24 %)	Chi-square test *p* = 0.745
Proximal segment	5 (16 %)	6 (24 %)	Chi-square test *p* = 0.745
AO/ASIF fracture type A	20 (62 %) (3 42-A1, 10 42-A2, 7 42-A3)	16 (64 %) (3 42-A1, 10 42-A2, 7 42-A3)	Chi-square test *p* = 0.565
Type 42-B2	12 (38 %)	9 (36 %)	Chi-square test *p* = 0.565
Days from fracture to op. mean ± SD (range)	16 ± 26 (6–110)	13 ± 18.7 (5–80)	*t* test *p* = 0.628

The gender, mean age, injury mechanism, location, type of fracture, and days between fracture and operation in the nails compressed by tube and conventional interlocking nails

The proximal compression tube nail system has two distal and one proximal locking screws 5 mm in diameter. Proximally, the nail has an oblong hole (5.5 mm wide and 20 mm long) and two distal round holes. Compression tube is 44 mm in length and 13 mm wide. As the central screw at the top of the compression tube system rotates, the compression tube moves downward pushing the proximal locking screw distally through the oblong hole of the nail creating a compressive force on the fracture site (Fig. 1).

In the proximal compression tube nail group (group one) (*n* = 32), 9-mm and 10-mm nails were used for 28 patients and four patients respectively. Nine patients required open reduction, while in the remaining 23 patients, close reduction was satisfactory. Whereas in the conventional interlocking nail group (group two) (*n* = 25), 9-mm and 10-mm nails were used for 22 patients and three patients respectively. Open reduction was performed for 13 patients. Reaming was applied for all patients in the two groups.

Patients were evaluated monthly. Union rate, locking screws failure, and nail migration were assessed and compared between the two groups. The functional results of patients were evaluated according to Johner–Wruh’s criteria (Table 2) [13].

**Table 2:** Criteria for evaluation of final results of tibial shaft fractures according to Johner and Wruh’s criteria

	Excellent	Good	Fair	Poor
Nonunion–amputation	None	None	None	None
Neurovascular disorder	None	Minimal	Moderate	Severe
Deformity Varus–valgus (°)	None	2–5	6–10	>10
Anteversion–recurvation (°)	0–5	6–10	11–20	>20
Rotation (internal–external (°)	0–5	6–10	11–20	>20
Shortening (mm)	0–5	6–10	11–20	>20
Mobility (knee) (%)	Normal	>80	>75	<75
Mobility (ankle) (%)	Normal	>75	>50	<50
Pain	None	Occasional	Moderate	Severe
Gait	Normal	Normal	Insignificant limp	Significant limp
Strenuous activity	Possible	Limited	Severely limited	None

We compared statistically the results using Chi-square tests (Fisher’s exact test) and *t* test. We defined the level of significant difference as *p* < 0.05.

## Results

In the fractures operated with the proximal compression tube nail group (1), no secondary surgeries were required. In the conventional interlocking nail group (2), the proximal locking screws were removed to dynamize the nail in six cases. The difference between the two groups was statistically significant (Chi-square (Fisher’s exact test) *p* = 0.005).

Union is considered when callus formation appears on three or four cortices on the AP and lateral X-rays. In group (1), the mean union time was 20 ± 2 range (16–24) weeks, whereas in group (2) the mean union time was 22 ± 2 range (17–27) weeks. The difference between the two groups was statistically significant with *p* = 0.001 (*t* test).

The functional results evaluated by the Johner–Wruh’s criteria in group (1) patients were recorded as, excellent in 19 patients (59.5 %), good in seven patients (22 %), fair in five patients (15.5 %), and poor in one patient (3 %), whereas the functional results recorded in group (2) patients were, excellent in 10 patients (40 %), good in eight patients (32 %), fair in four patients (16 %), and poor in three patients (12 %) (Table 3). There was no statistically significant difference between the two groups regarding the functional results.

**Table 3:** Comparison of functional results of tibial fractures operated by nails compressed by tube and conventional interlocking nails according to Johner and Wruh’s criteria

	The nail compressed by tube (*n* = 32)	Interlocking nail (*n* = 25)	*p* value Pearson Chi-square test
Excellent	19 (59.5 %)	10 (40 %)	*p* = 0.140
Good	7 (22 %)	8 (32 %)	*p* = 0.140
Fair	5 (15.5 %)	4 (16 %)	*p* = 0.140
Poor	1 (3 %)	3 (12 %	*p* = 0.140

Nonunion was recorded in only one case in group (1), while there were three nonunion cases recorded in group (2). However, this difference was not statistically significant according to the Chi-square (Fisher’s exact test) test (*p* = 0.079).

In group (1), locking screw failure and nail migration were not recorded in any case. However, in group (2), two proximal locking screw failure (screw bending) and associated proximal nail migration about 8 mm were recorded. The difference was not statistically significant (*p* = 0.18).

## Discussion

In the conventional interlocking nail group, additional surgical interventions were required for nail dynamization, while in the proximal compression tube group, there was no additional operation required.

The oblong hole in the proximal compression tube nail system is 20 mm in length which offers an enough space after compression for self-dynamization. Even if there is bone resorption on both fracture ends, self-dynamization (i.e., telescoping) feature will always ensure contact between fracture ends, which leads to the prevention of secondary operations like dynamization.

A period of 2 weeks earlier union rate was recorded with proximal compression tube nails. In proximal compression tube nails, self-dynamization effect (i.e., telescoping) feature exerted by the 20-mm oblong hole results in a continuous contact between fracture ends which may ensure a faster union time rate even if there is a bone resorption at the fracture site, whereas in the conventional interlocking screw group, fracture site bone resorption may cause a delay in union due to the lack of self-dynamization effect.

Locking screw failure with proximal compression tube nails was not documented; however, two locking screw bending failures with the conventional interlocking nails had been recorded. We assumed that the main reason for this is the self-dynamization effect feature of the 20-mm oblong hole in addition to the continuous contact between the fractured edges. The continuous contact effect between the fractured edges may decrease the bending stress on locking screws by sharing the axial loads. Higher locking screw failure rates had been reported for the conventional interlocking nails [8–10]. The locking screw bending failure had been reported to occur intraoperatively during nail compression by central screws [3].

Nail migrations to the knee joint causing recurrent anterior knee pain had been reported by some studies with the conventional interlocking nails [11, 12, 14, 15]. Nail migration from bone causing damage to joint and tendons had been documented in two cases in the conventional interlocking nail group. However, nail migration had not been encountered in the proximal compression tube nail group.

In conventional nails compressed by the central screw system, as compression is applied intraoperatively, nail migrates proximally to the knee joint. For this reason, the nail should be implanted lower and more distal to the tibial plateau. But when more compression is needed than the estimated amount, the proximal tip of the nail migrates proximally toward the knee joint. On the contrary when less compression is required, the nail tip remains buried in the proximal tibial which in turn may create difficulties in revision and removal surgeries [11, 12]. However, proximal compression tube nail system does not migrate proximally during compression stage due to the nail length reduction during compression procedure. No matter how much compression is required, the proximal end of the nail remains fixed at the same level of insertion.

The proximal locking screw in the proximal compression tube nail system has more bending resistance due to the two separate point contact effects of the tube diameter, while in the conventional nail systems, compression is exerted by only a central screw with a single point contact, which in turn increasing the bending stress effect of the proximal locking screw, thus leading to a high rate of screw failure.

We assumed that the proximal compression tube nail is not appropriate to be used in spiral tibial shaft fractures (AO/ASIF 42-A1) and bending wedge fractures (AO/ASIF 42-B2) except for fractures that can achieve perfect reduction.

However, the weakness of this study is being a retrospective with a small patient population, and further prospective studies should be conducted with a larger patient population.

## Conclusion

The proximal compression tube nails are more convenient than conventional interlocking nails for transverse and oblique tibial shaft fractures (AO/ASIF 42-A2, 42-A3) without the need for additional dynamization surgeries, with a less complication rates of nonunion, proximal locking screw failure, and proximal nail migration.
